# An Open-Source, Standard-Compliant, and Mobile Electronic Data Capture System for Medical Research (OpenEDC): Design and Evaluation Study

**DOI:** 10.2196/29176

**Published:** 2021-11-19

**Authors:** Leonard Greulich, Stefan Hegselmann, Martin Dugas

**Affiliations:** 1 Institute of Medical Informatics University of Münster Münster Germany; 2 Institute of Medical Informatics Heidelberg University Hospital Heidelberg Germany

**Keywords:** electronic data capture, open science, data interoperability, metadata reuse, mobile health, data standard, mobile phone

## Abstract

**Background:**

Medical research and machine learning for health care depend on high-quality data. Electronic data capture (EDC) systems have been widely adopted for metadata-driven digital data collection. However, many systems use proprietary and incompatible formats that inhibit clinical data exchange and metadata reuse. In addition, the configuration and financial requirements of typical EDC systems frequently prevent small-scale studies from benefiting from their inherent advantages.

**Objective:**

The aim of this study is to develop and publish an open-source EDC system that addresses these issues. We aim to plan a system that is applicable to a wide range of research projects.

**Methods:**

We conducted a literature-based requirements analysis to identify the academic and regulatory demands for digital data collection. After designing and implementing OpenEDC, we performed a usability evaluation to obtain feedback from users.

**Results:**

We identified 20 frequently stated requirements for EDC. According to the International Organization for Standardization/International Electrotechnical Commission (ISO/IEC) 25010 norm, we categorized the requirements into functional suitability, availability, compatibility, usability, and security. We developed OpenEDC based on the regulatory-compliant Clinical Data Interchange Standards Consortium Operational Data Model (CDISC ODM) standard. Mobile device support enables the collection of patient-reported outcomes. OpenEDC is publicly available and released under the MIT open-source license.

**Conclusions:**

Adopting an established standard without modifications supports metadata reuse and clinical data exchange, but it limits item layouts. OpenEDC is a stand-alone web app that can be used without a setup or configuration. This should foster compatibility between medical research and open science. OpenEDC is targeted at observational and translational research studies by clinicians.

## Introduction

High-quality data are crucial for obtaining medical research results [1] and successful machine learning applications [2]. To collect and manage structured data in digital format, researchers can use computer programs called electronic data capture (EDC) systems [3]. There is a consensus that EDC leads to improved data quality as well as cost and time efficiency compared with paper-based methods. Direct data entry at an investigator site reduces the probability of transcription errors [4,5]. By detecting missing fields and data type and range violations, EDC systems offer data validation at the time of entry instead of days or even weeks later [6]. Finally, real-time access allows information managers to continuously monitor the collection process [7], review and analyze data in real time [8], and improve the feedback loop with local investigators [9].

Data exchange and data compatibility are two of the most important areas in medical research. However, proprietary or customized data formats used by EDC vendors render this endeavor a major point of concern. As a result, incompatible electronic case report form (eCRF) data structures impede data integration and analysis from different sources and, hence, the full potential of captured information [10]. A system that fosters compatible data structures through standardization could pave the way toward more open science to improve scientific understanding and enhance patient care [11]. In addition, EDC remains underused despite its benefits [6]. The configuration, maintenance, and financial requirements of typical EDC systems are common obstacles for dissemination and may prevent small-scale studies to profit from digital data collection [12]. Owing to these shortcomings of professional EDC systems, practitioners frequently resort to inappropriate software for data collection, such as general-purpose spreadsheet applications [13]. However, these are considered inflexible, insecure, and complicate data compatibility [14,15]. In addition, they do not provide an audit trail to trace data changes.

In this study, we describe the development process of OpenEDC to address the aforementioned issues. OpenEDC is an EDC system based on the results of a systematic requirements analysis. To the best of our knowledge, this study makes two unique contributions. First, OpenEDC is entirely based on the regulatory-compliant and internationally accepted Clinical Data Interchange Standards Consortium (CDISC) Operational Data Model (ODM) standard [16]. It is used without modifications to allow for fully standardized metadata and research data import and export. This facilitates metadata reuse and clinical data exchange, whereas most EDC systems use custom or highly modified formats. Second, a client-based web approach allows researchers worldwide to use OpenEDC without installation or configuration needs. Therefore, it is a valuable alternative to spreadsheet applications. An optional server enables distributed data capture and access whenever necessary. In addition, OpenEDC focuses on cross-platform support for desktop computers and mobile devices to allow the collection of increasingly important patient-reported outcomes. We made OpenEDC publicly available [17] and released it under the MIT open-source license [18].

The remainder of this paper is structured as follows: the Methods section outlines the requirements analysis and evaluation process of OpenEDC. The Results section gives an overview of the identified requirements, the resulting software, and its evaluation outcomes. The contributions, limitations, and future work are discussed in the Discussion section.

## Methods

### Requirements Analysis

OpenEDC was developed within the context of a large-scale medical register project for chronic diseases. For an intended period of more than 10 years, most German university hospitals were to collect patient-reported outcomes and medical routine data with tablet and desktop computers. During the system selection process, however, the shortcomings of the present EDC systems became apparent. On the basis of the register’s long-lasting nature, an ideal system was open source so that it could be maintained in the future without manufacturer dependency or insecure licensing conditions. Being open source would also reduce the risk of unaffordable expenses once the funding of the register might have expired. In addition, standardized metadata import was requested as we had the most eCRFs in the standardized CDISC ODM format. This would allow us to use these methods without time-consuming and error-prone manual transmission. A standardized system would also allow us to export metadata or captured clinical data in a reusable, interoperable, and nonproprietary format in the future. Finally, an easy-to-use and network-independent support for mobile devices was necessary for data collection at the participating sites.

In addition to the project-specific demands, we performed a literature-based requirements analysis to ensure the applicability of OpenEDC in a wide range of research projects. This analysis included the following three steps: first, a literature search revealed the EDC requirements stated by both academics and public bodies. Keywords for searching in the academic repositories PubMed and ScienceDirect were *electronic data capture*, *EDC system*, *digital data collection*, *data management*, and *electronic case report form*. For ScienceDirect, we also added the keywords *clinical trial, health study*, and *medical research*. We scanned the top 60 search results for each query. The selection criteria were thematization of EDC-related functionality, low to moderate resource settings, and generalizability (ie, very specific use cases were excluded). After identifying appropriate titles, reading abstracts, and recursively evaluating references, 18 publications were chosen for in-depth analysis (Table 1). The identified publications can be categorized into review studies that evaluated EDC implementation and use (n=8), original reports of trials that used EDC (n=6), and descriptions of EDC system development (n=4). Second, 2 team members with experience in several EDC projects prioritized the identified requirements. This prioritization happened amid the aforementioned internal register requirements and therefore influenced prioritization (see the Discussion section). Third, the International Organization for Standardization/International Electrotechnical Commission (ISO/IEC) 25010 norm and its software quality model were used to categorize the prioritized requirements [19]. The resulting requirements are listed in Table 1.

**Table 1:** Main requirements and subrequirements of OpenEDC. Subrequirements are based on commonly stated electronic data capture requirements in the literature. The main categorizing requirements and their definitions originate from the ISO/IEC 25010 norm [19].

Requirement	Definition	Subrequirements
Functional suitability	Product or system provides functions that meet stated and implied needs when used under specified conditions	Design [13,20] and reuse [14,21] of metadata Capture and store clinical data [15,20-23] Form completion tracking [3,24,25] Field validations (edit checks) [8,13-15,20,21,23,25,26] Conditional fields (skip patterns) [8,13,15,23,24,26] Multicentric (multisite) studies [5,13,14,21] Longitudinal studies (with defined events) [13,24,26] Multilingual forms [9,24]
Availability	System, product, or component is operational and accessible when required for use	Open source [4,8,12-14,20,22,23] Minimal setup and configuration [4,6,12] Distributed (near) real-time access [5,8,14,21,22,24] Cross-platform (mobile device support) [9,13,15,26,27] Offline-capable [15,26,27]
Compatibility	Product, system, or component can exchange information with other products, systems, or components	Standard-compliant import and export of metadata and clinical data [4,6,13,14] Semantic annotation (medical coding) of items [12,14,20]
Usability	Product or system can be used by specified users to achieve specified goals with effectiveness, efficiency, and satisfaction in a specified context of use	Ease of use (user-friendly) [4-6,13-15,21] Medical staff and patient accessibility [4,5]
Security	Product or system protects information and data so that persons or other products or systems have the degree of data access appropriate to their types and levels of authorization	Authentication and authorization (user rights and roles) [13,20,21] Encrypted data storage and transmission [14,15,22,26] Audit trail [3,8,12,14,20-22]

An iterative waterfall model was used to implement the identified requirements [28]. Fundamental and technological requirements were used to design the software architecture. The architecture determines the overall structure, programming language, and supported computer platforms. After architecture design, the most basic and EDC-inherent functions were implemented first, while specific functions were subsequently added. For example, as EDC systems typically follow a metadata-driven approach, that is, allow data capture within the constraints of previously designed eCRFs, the metadata design module was implemented first, followed by modules for data capture and data export. After implementing the key requirements and internal testing, we evaluated OpenEDC to receive the first feedback from potential users. This linear sequence of requirements analysis, design, implementation, and evaluation will be further used to iteratively add secondary functions during the system life cycle.

### Evaluation

OpenEDC was evaluated against the identified requirements. However, whereas most of these requirements can be evaluated qualitatively in an absolute sense, that is, achieved or not achieved, usability is perceived subjectively and difficult to generalize [29]. To perform a generalizable and comparable assessment of usability, OpenEDC was evaluated using the system usability scale (SUS) [29]. The SUS is a “10-item scale giving a global view of subjective assessments of usability” that has been used in numerous research projects [30]. After defining a system-related task, 16 participants completed the task and answered the 10-question survey. The participants were recruited via an institution-wide mailing list. At the time of evaluation, none of them were involved in the OpenEDC project. All survey responses resulted in an average score ranging from 0 to 100, estimating the usability of the system. A score above 70 denotes a user-friendly system [31].

Before the actual task, a video was shown to the users to explain the main functionalities of OpenEDC. This video is openly available and can be consulted by prospective users as well [32]. Subsequently, the users were asked to accomplish the following typical EDC tasks using the desktop version of OpenEDC. First, they were asked to reuse and modify case report forms using the metadata design module. For the designed forms, they were prompted to capture data for multiple simulated patients. In the third step, they were instructed to export the collected data while verifying that all data were correctly included in the export file. Finally, the users were invited to answer the SUS survey and 2 open questions about what they liked or disliked in particular. OpenEDC was used to capture all participants’ answers remotely.

## Results

### Requirements Analysis

#### Functional Suitability

A fundamental requirement for EDC systems is the support for metadata design [13,20]. The ability to reuse defined metadata elements can facilitate the design process [14,21] (also see the *Compatibility* section). Data element types and range checks enable an EDC system to evaluate data entries in real time and report violations. This is called real-time field validation [14] (plausibility [15] or edit checks [25]), and it increases the data quality significantly. Conditional fields [15] (or skip patterns [26]) can reduce form completion time and improve data quality by showing or hiding items based on prior inputs. After data entry, form completion tracking enables investigators to recognize which form has been completed for which subject [25]. When research is conducted at geographically dispersed sites, that is, research or clinical centers, it is important to keep track of which subject has been included at which site [5]. Typically, local investigators can access only subject data from one site, in contrast to central information managers [14]. To support longitudinal studies, the system needs to allow the definition of events or visits [24]. Finally, medical research studies may include patients with different demographic backgrounds that require multilingual forms to capture patient-reported outcomes [9].

#### Availability

Availability is frequently stated as an important property or, if absent, the reason for the limited dissemination of EDC systems [4,6,12]. However, it was noted that “open-source EDCs have the potential of increasing and improving public health research activities and raising academic standards because of their availability” [22]. A community that forms around open-source software can collaboratively enhance software quality and increase the probability of its long-term existence. However, even when a system is open source, implementation and maintenance requirements can hamper the availability of software [12]. A web server has been stated as an essential resource for supporting an EDC system [14], together with challenging installation, customization, and configuration requirements [22]. A system that can be used without assistance from potentially expensive information technology specialists could prevent practitioners from resorting to inappropriate spreadsheet applications [14,15]. Once an EDC system is established, distributed access usually allows remote data entry and real-time monitoring, which is particularly important for multicentric studies [22]. Moreover, multiple computer platforms may be used within one research study, such as tablets for data capture and desktop computers for data management [13]. A related subrequirement is offline capability. An active internet connection cannot always be guaranteed, such as in rural research settings or owing to hospital walls that shield mobile network signals [26].

#### Compatibility

Standardized data formats and coding of data elements can foster data compatibility [33]. Standardized data formats result in syntactic compatibility and facilitate integration and interpretation of clinical research data without the need to apply a proprietary data format [34]. Moreover, it allows the reuse of metadata elements from previous subject-related studies, leading to a simplified metadata development process and data compatibility at the design stage [10]. We agreed to support the regulatory-compliant and well-established CDISC ODM standard, which is “a vendor-neutral, platform-independent format for exchanging and archiving clinical and translational research data, along with their associated metadata, administrative data, reference data, and audit information” [16]. It is the fundamental part of Define-XML [35], which is included in the United States Food and Drug Administration Data Standards Catalog [10,36]. See the Discussion section for comparison with other medical data standards. Finally, the annotation or medical coding of data elements facilitates semantic compatibility. A unique semantic code from a terminology such as the unified medical language system assigned to an item allows unambiguous mapping independent of language or wording [37].

#### Usability

It was reported that “the lack of a simple, intuitive, and user-friendly EDC system is noteworthy” [14]. Moreover, Franklin et al [13] stated that in “a 2-year qualitative evaluation we found that the importance of ease of use and training materials outweighed number of features and functionality” of EDC systems. As ease of use is also considered to positively impact adoption, data quality, and overall success of EDC initiatives [14], it was added to the list of requirements. In addition, patient-reported outcomes have recently become more relevant to medical research [1]. Data entry for both investigators and patients is regarded as a desirable characteristic of an EDC system [4]. As a result, medical staff and patient accessibility are formulated requirements to enable investigators to capture both routine data and patient-reported outcomes.

#### Security

Regulatory bodies frequently address the data protection and privacy measures of computerized systems in clinical trials. The General Data Protection Regulation (GDPR) of the European Union, for example, became enforceable in all European Union member states in May 2018 [38]. It covers the personal data of all European Union residents [39] and is considered a driving force for international data protection standards [40]. GDPR demands that personal data, and health data as a special category of personal data in particular, is “processed in a manner that ensures appropriate security of the personal data, including protection against unauthorised or unlawful processing” and suggests the “encryption of personal data” [38]. Authentication and authorization, as well as encrypted storage and transmission of data, were added to the system requirements. In addition to the GDPR, national regulations specifically targeting clinical EDC systems exist. Title 21, Part 11 of the Code of Federal Regulations, for example, issued by the Food and Drug Administration, requires EDC systems to maintain a continuous audit trail [41]. In fact, an audit trail is an often stated and essential accountability requirement for every EDC system [12]. It allows investigators, sponsors, and public bodies to seamlessly trace any changes made to electronic records, including time and author.

### Design and Implementation

#### CDISC ODM Data Schema

OpenEDC [17] is based on the CDISC ODM standard. Although initially targeted for allowing the reuse of metadata, we capitalized on the standard's data schema to achieve several other identified requirements. This was possible because this standard not only provided guidance in defining metadata but also in storing associated clinical and administrative data. As a result, OpenEDC can be seen as an editor or user interface for CDISC ODM documents and facilitates the application of this standard.

The CDISC ODM provides the groundwork for achieving the following system requirements. From the metadata perspective, events are at the highest hierarchical level with subordinate forms to allow the representation of longitudinal studies. Descriptive or interrogative texts can be defined as multiple translations for multilingual projects. Most frequently, these texts are assigned to data items for which data are to be collected. Data items have data types and may also have specified value ranges to enable real-time field validation. Moreover, items can be dynamically hidden to support conditional fields. Item definitions can be further referenced and reused in other locations. To complement the data schema for the remaining *functional suitability* requirements, the CDISC ODM specifies structures for subject-related data storage, including references to form completion states and site information.

All of the specifications were implemented and internally used by OpenEDC. This results in fully standard-compliant imports and exports of both metadata and clinical research data. In addition, the CDISC ODM enables the annotation of data items with an arbitrary number of semantic codes. These features constitute the *compatibility* requirements. Finally, the CDISC ODM provides guidance for implementing an audit trail, including author information and timestamps for data modifications. This specification was used to partially address *security* requirements.

#### Client-Based Web App

The *availability* requirements are addressed using a client-based web app. Client-based refers to a static web app that includes all business logic and persistence. It allows researchers to design or reuse eCRFs and capture clinical data without the assistance of information technology specialists or the need for a web server. Moreover, web technology supports the development of cross-platform apps running on all devices using a web browser. Users are not required to install or configure any external software that is important in a clinical setting, where users do not have administrative rights on a standard computer. OpenEDC is a progressive web app that can be installed as a stand-alone system on desktop computers, tablets, and smartphones [42]. In addition, a service worker enables offline data capture that is needed in regions without consistent and reliable internet connections [43]. We decided not to use a third-party JavaScript framework to reduce long-term functional dependencies and developed OpenEDC by using modern browser technologies such as web components [44] and modules [45].

**Figure 1:**
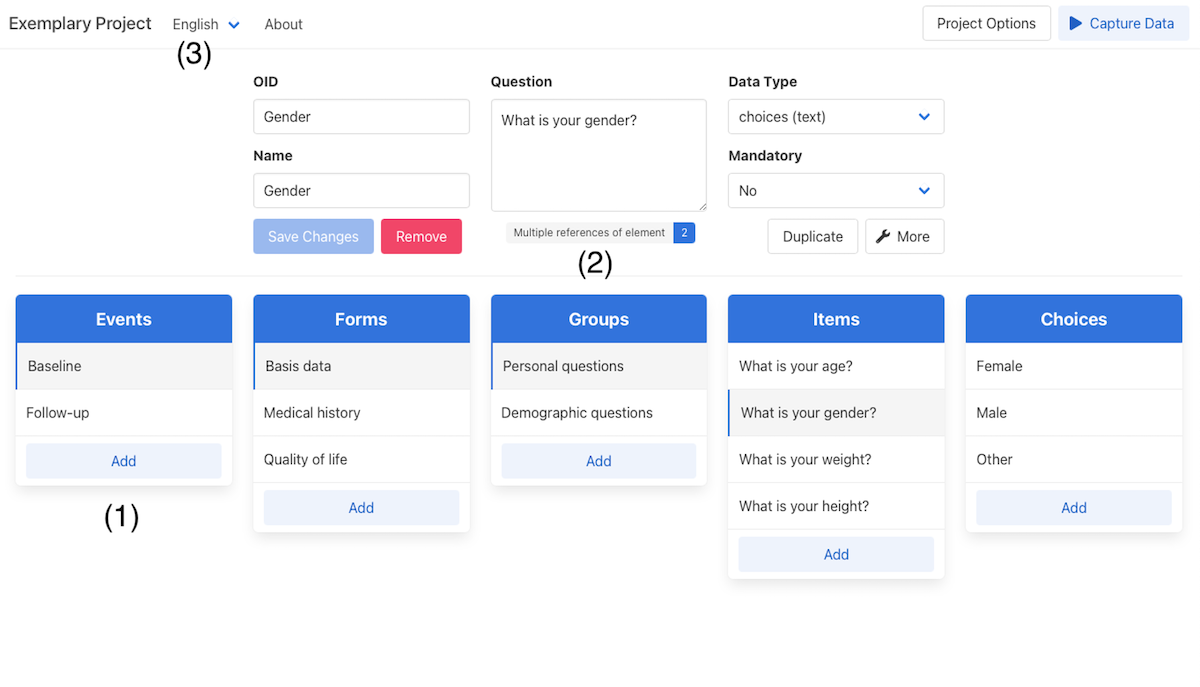
User interface of the metadata design mode. The hierarchical order of metadata elements is represented by the centered column view (1). By means of a referencing system, electronic case report forms (eCRFs) can be reused entirely or partially (2). The language of eCRFs can be changed with the drop-down at the top left (3).

**Figure 2:**
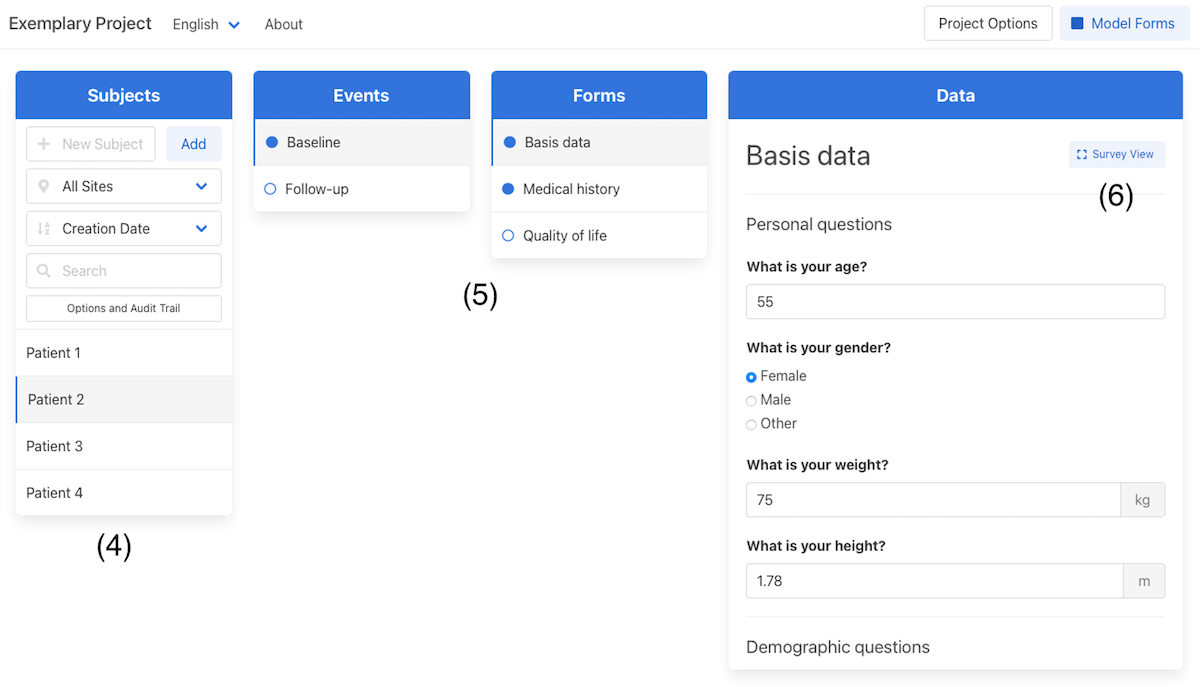
User interface of the clinical data capture mode. Subjects can be managed with the left column where an audit trail can be accessed as well (4). Filled or empty circles in the 2 center columns indicate whether an event or form has been completed (5). A survey view button within the right electronic case report form column switches to a mode for patient-reported outcomes (6).

We implemented a simple user interface to address the *usability* requirements. The interface is structured into 2 modes, from which one is used for metadata design and the other for clinical data capture. Modes can be switched at any time to see the rendered form previews during the metadata design phase. We further integrated known concepts such as drag-and-drop, keyboard navigation, and a hierarchical, column-based file structure. A special mode was implemented to support the collection of patient-reported outcomes. Once activated, unnecessary user interface elements become hidden and access to data from other patients is prevented. The user interface of both modes on a desktop computer is shown in Figure 1 (metadata design) and Figure 2 (clinical data capture).

#### Optional Client-Server Architecture

**Figure 3:**
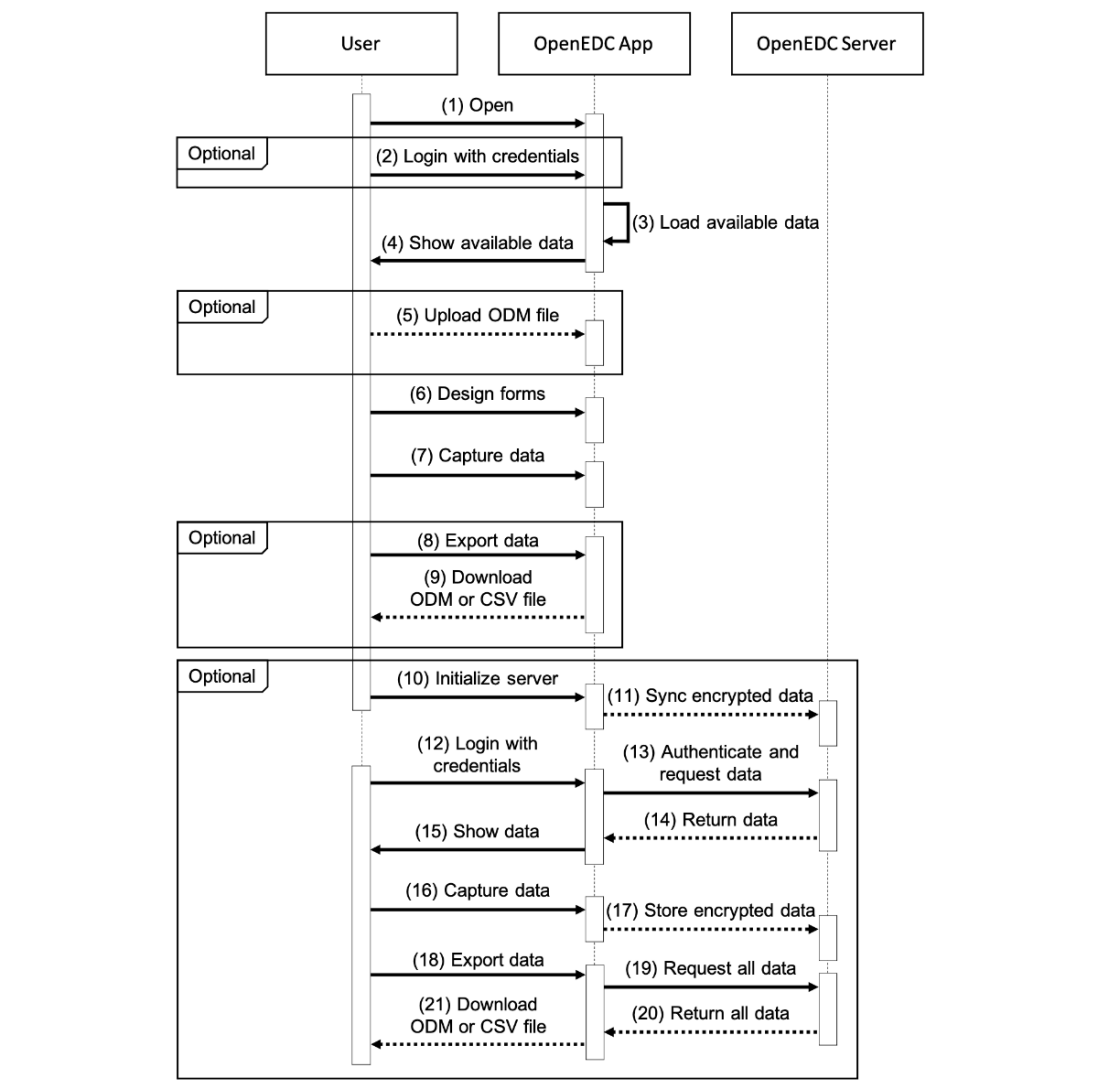
Sequence diagram of a typical use scenario with OpenEDC. In this example, the stand-alone OpenEDC web application is used to design electronic case report forms and capture data. A Clinical Data Interchange Standards Consortium Operational Data Model file can be uploaded to reuse metadata or import clinical data. Optionally, the user can initialize an empty OpenEDC server with locally stored data. This enables the user to set up a multiuser system and conduct multicentric research studies. EDC: electronic data capture; ODM: operational data model.

To accomplish the *availability* requirement of distributed real-time system access for multiuser and multicentric research studies, we developed an optional OpenEDC server [46]. A connection to the server can be established even after the data are captured locally using the stand-alone OpenEDC web app. All data are synchronized with the host server. From this time on, new user accounts can be created, and collaboratively captured data are centrally stored on the OpenEDC server. Figure 3 shows a sequence diagram of this usage scenario.

The server provides authentication and authorization services to address the remaining *security* requirements. Clients can authenticate against a representational state transfer application programming interface [47]. When authenticated, the server verifies that a user has the required authorization to access the requested application programming interface end point. End points are available for reading, storing, and removing metadata, clinical subject data, or administrative data. Each end point enforces different user rights. All data is further transferred and stored encrypted. The OpenEDC web app enforces an encrypted https over a transport layer security connection. In addition, all data are encrypted before transfer from the client to the server and can only be decrypted by an authorized client when received back from the server. This ensures that even people with logical or physical server access cannot read data without permission. Local password encryption can also be used to encrypt data when the OpenEDC web app is not connected to a server.

### Evaluation

Usability is a subjectively perceived characteristic of a particular context and user [29]. The SUS was used to estimate the usability of OpenEDC. In total, 16 persons were asked to participate in the test, of which 50% (8/16) were women and 50% (8/16) were men. All participants were digital health or biomedical domain experts with an average work experience of 5.3 years (SD 3.1 years) in medical research projects.

OpenEDC achieved a mean usability score of 83.1 (SD 9.6) out of 100. Men rated it slightly lower than women with an average score of 82.5 (SD 11.7) compared with 83.8 (SD 7.6). Two additional open questions were answered by 75% (12/16) of the participants. They provided very heterogeneous suggestions for improvement, with most being related to the user interface and few to functionality. Interface-related suggestions were shortcut buttons for frequently used functions, more noticeable highlighting of inputs with implausible data, and a larger visual difference between the metadata and clinical data view. Introducing simple statistics for data completeness and patient enrollment, labeling conditionally unavailable items in the CSV export, and improving support for older browsers were suggestions related to functionality. Most participants stated that they liked the clear user interface and the performance of the system.

## Discussion

### Principal Findings

This paper describes the implementation process of OpenEDC, an open-source and standard-compliant EDC system for medical research. We conducted a requirements analysis to identify the academic and regulatory demands for digital data collection. After implementation, we performed a usability evaluation to obtain feedback from the users. OpenEDC achieved a mean usability score of 83.1, which can be considered user-friendly [31]. OpenEDC is available worldwide without installation or configuration requirements. It focuses on cross-platform support for desktop and mobile devices to allow the collection of increasingly important patient-reported outcomes.

### Strengths and Limitations

OpenEDC is based on the CDISC ODM standard, yielding several advantages. Metadata and clinical research data can be imported and exported without constraints in a nonproprietary format and without vendor lock-in effects. Investigators may also download eCRFs from public metadata registries, such as the Portal of Medical Data Models [48], to swiftly create databases for data capture. Therefore, we hope to encourage the reuse of metadata, foster compatibility of medical research, and ultimately support open science [11]. In contrast, the CDISC ODM is relatively limited when it comes to the visual representation of items. For example, multiple-choice questions need to be implemented as lists of Boolean items. Moreover, it is impossible to uniformly distinguish between single-choice items rendered as radio buttons or as a drop-down list or to label multiple items with the same predefined choices as a Likert scale. Other systems that support the CDISC ODM often work around this limitation by extending or modifying the standard. However, this can render a system incompatible with other systems. Research Electronic Data Capture (REDCap) [21], for example, imports and exports REDCap-specific CDISC ODM files but often fails when attempting to import standard-compliant files. Currently, OpenEDC favors standard conformance over nonspecified input types.

Other standards exist for exchanging metadata and clinical research data. For example, Fast Healthcare Interoperability Resources (FHIR) from Health Level 7 (HL7) is increasingly adopted to exchange electronic health records and other information in the medical domain [49]. Resources constitute the fundamental building block and are also available for eCRF metadata and their associated clinical data, called Questionnaire and Questionnaire Response, respectively [50]. Although these are rather unspecific by default, the structured data capture (SDC) implementation guide targets improved interoperability [51]. In contrast to the CDISC ODM, however, the HL7 FHIR SDC does not provide a holistic archive format, including users, sites, audit trail records, and their relationships to captured information. Moreover, it does not support the definition of longitudinal events or multiple languages by default. These limitations made HL7 FHIR alone unsuitable for the requirements of the present EDC system. However, as it is widely used to exchange more discrete parts of data, we prepared to implement support for the import and export of HL7 FHIR SDC Questionnaire and Questionnaire Response resources at the time of writing. A similar approach can be adopted for ISO/IEC 11179 [52] and ISO/TS (International Organization for Standardization Technical Specification) 21526 [53]. Both are metadata standards, with the ISO/TS 21526 explicitly targeting the health care sector [53]. However, as they do not provide a specific data format to support the syntactic compatibility of information [54], integration is currently not prioritized.

OpenEDC is publicly available for the creation of local studies. The app is available via the web for desktop and mobile devices, whereas data storage occurs locally and encrypted. This architecture allows researchers to benefit from metadata-driven digital data collection without an information technology department, web server configuration issues, or device constraints. In addition, it leaves data sovereignty to the investigator, rather than a third-party infrastructure or server provider. While this approach offers advantages in terms of flexibility, it also has some drawbacks. It is generally helpful to have a dedicated computer scientist who can make educated decisions about data security, data backup, and metadata design concerns. Moreover, it may be beneficial for a study’s sustainability to have a contact person for technical problems and issues. However, it is worth noting that an information technology specialist can still be employed when using OpenEDC. In particular, when an OpenEDC server must be configured, for example, for projects with multiple users and sites, knowledge in setting up a web server is important. In our opinion, OpenEDC’s architecture is particularly useful for investigator-initiated studies and enables researchers to set up and test databases before information technology support and infrastructure investments have to be made.

### Comparison With Prior Work

Other EDC systems also exist. One of the most frequently used EDC systems is REDCap [21]. REDCap provides various functions that are not present in OpenEDC, such as an extensive admin server dashboard, support for surveys that can be sent via a link to participants, and a module for randomization. While a systematic comparison is beyond the scope of this work, there are some aspects in which OpenEDC has advantages. For example, although REDCap is free to use, it is strictly licensed and not open-source, requires a web server, and is not standard-compliant, as it uses a customized CDISC ODM syntax. It is worth noting that open-source EDC systems also exist. Examples include the OpenClinica Community Edition [55], Open Data Kit (ODK) ecosystem [56], the Rare Disease Registry Framework [57], and the Open-Source Registry System for Rare Diseases [58]. OpenClinica and ODK are established systems with functionalities that are absent in OpenEDC. For example, OpenClinica provides double data entry and a query management system. ODK provides more input types, such as sliders, as well as widgets for image capturing and drawing. However, OpenClinica Community Edition requires a web server, form design via Microsoft Excel, and is not suitable for smartphones or tablet computers. On the other hand, data capture using ODK is designed only for Android mobile devices. While OpenClinica and ODK are multipurpose EDC systems, the Rare Disease Registry Framework and Open-Source Registry System for Rare Diseases specifically target registries for rare diseases. Similar to OpenEDC, the 2 systems address technically underresourced settings and foster metadata reuse. However, both lack offline mobile device support and a standardized export of metadata and clinical research data.

### Future Work

Future work is necessary. The main objective was to ensure the applicability of OpenEDC to a wide range of research projects. However, literature-based requirements analysis was influenced by the demands of a large-scale medical register. Rarely mentioned requirements were not included if they were not required by the internal project. Examples of rarely mentioned but deferred demands are integrated query management as well as document storage and report functionalities. In addition, although OpenEDC complies with relevant laws and regulations, including 21 Code of Federal Regulations Part 11 and GDPR, a computer system validation required for interventional trials has not yet been conducted. Validating an EDC system is also trial-specific and requires activities by the investigator or sponsor. Currently, we see OpenEDC’s distinct advantages for observational and translational research studies by clinicians rather than commercial clinical trials. We hope it is a valuable first step toward an openly available, standard-compliant, and mobile EDC system. We plan to develop OpenEDC further and use it in prospective studies. To expand the support for varying study protocols, unavailable functions stated earlier should be added. We hope for contributions from the research community, as we have published OpenEDC under the MIT open-source license.

### Conclusions

We showed that it is possible to develop an EDC system for use without upfront investment and preservation of data sovereignty. The primary focus was on standard compliance to foster metadata reuse, interoperable research data, and open science. Future work is necessary to extend the system’s functionality and prove its robustness in large-scale studies. OpenEDC is publicly available and released under the MIT open-source license.

## Abbreviations

CDISC: Clinical Data Interchange Standards Consortium
eCRF: electronic case report form
EDC: electronic data capture
FHIR: Fast Health care Interoperability Resources
GDPR: General Data Protection Regulation
HL7: Health Level 7
ISO/IEC: International Organization for Standardization/International Electrotechnical Commission
ODK: Open Data Kit
ODM: operational data model
REDCap: Research Electronic Data Capture
SDC: structured data capture
SUS: system usability scale
